# Validation of SYBR Green based quantification assay for the detection of human Torque Teno virus titers from plasma

**DOI:** 10.1186/1743-422X-10-191

**Published:** 2013-06-11

**Authors:** Anuj Kumar Tyagi, Amandine Pradier, Odile Baumer, Chakradhara Rao S Uppugunduri, Patricia Huezo-Diaz, Klara M Posfay-Barbe, Eddy Roosnek, Marc Ansari

**Affiliations:** 1 Department of Pediatrics, Onco-Hematology Unit, Geneva University Hospital, Rue Willy Donzé 6, 1211, Geneva, Switzerland; 2 CANSEARCH Research Laboratory, Geneva Medical University, Geneva, Switzerland; 3 Division of Hematology, Geneva University Hospital and Medical School, Geneva, Switzerland; 4 Department of Genetic and Laboratory medicine, Geneva University Hospital, Geneva, Switzerland; 5 Infectiology Unit, Department of Pediatrics, Geneva University Hospital, Geneva, Switzerland

**Keywords:** SYBR Green, Real-time PCR, Human torque teno virus

## Abstract

**Background:**

Quantification of titers of ubiquitous viruses such as Torque teno virus (TTV) that do not cause clinical symptoms might be helpful in assessing the immune status of an individual. We hereby describe the validation of a SYBR Green-based TTV quantification method for plasma samples.

**Methods:**

Plasmids with TTV specific inserts were used for preparing standards and absolute quantification of TTV was performed using SYBR Green methodology. The method was assessed for its accuracy and precision (intra and inter-day) on four non-consecutive days. TTV was also quantified from plasma samples of 20 healthy volunteers and from 30 hematopoietic stem cell transplant (HSCT) recipients.

**Results:**

The assay was specific and showed satisfactory efficiency (82.2%, R^2^=0.99) with the limit of quantification defined as 100 copies per reaction. The assay had good precision (inter and intra-day coefficient of variation in cycle threshold (C_T_) < 4%) and accuracy (100 ± 10%) in the range of 100 to 10^10^ copies/reaction. We found TTV loads ranging from 2.5 – 4.07 log copies/mL of plasma with C_T_ (mean ± SD) of 33.8 ± 1.77 in healthy individuals and 2.06 – 8.49 log copies/mL of plasma with C_T_ (mean ± SD) of 24.3 ± 1.04 in HSCT recipients.

**Conclusion:**

SYBR Green-based q-PCR assay combines simplicity with satisfactory sensitivity and may be suitable for monitoring the immune status of transplant recipients, where TTV loads over time may serve as a marker for immune reconstitution in human plasma samples.

## Background

Torque teno virus (TTV), classified into the family *anelloviridae*, genus *alphatorquevirus*, was first described in a patient with non-A-E hepatitis [1,2]. TTV is a non-enveloped, single-stranded, circular DNA virus present in plasma of >90% of individuals, regardless of geographical origin, age or health status [2,3]. Viral titers in the plasma may reflect the individual’s immune status, since immunocompromised patients harbor high loads of TTV [4]. This approach can be used to estimate immune recovery in hematopoietic stem cell transplant (HSCT) recipients by monitoring TTV titers after transplantation [5].

TTV DNA has a total genomic length of approximately 3.8 kilobases [6-9] and contains two large-open reading frames (ORF1 and ORF2) and several smaller ORFs [3]. TTV exhibits a wide range of sequence variability, with at least 38 TTV genotypes and forms five distinct phylogenetic groups [10-13]. The conserved ORF-2 region allows the design of primers expected to amplify most strains of TTV [14].

Several previously described quantification methods for human TTV [15-17] are based on TaqMan technology, which may be less suitable for quantification of highly variable viruses such as TTV. SYBR Green-based PCR with primers annealing to more conserved regions may be preferable. In the present study, we report the validation of SYBR Green based quantification assay for routine use by using a set of primer pairs targeted for amplifying a well-conserved sequence of ORF-2 [14].

## Results and discussion

**Figure 1:**
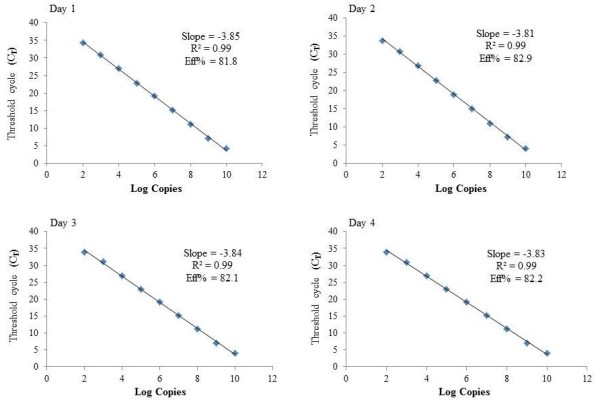
**SYBR Green based standard curve from two independent 10 fold serial dilutions of plasmid standards.** Standard curve was plotted in the sample plasmid on the x-axis and threshold cycle (C_T_) on the y-axis. The x-axis represents human TTV in 10-fold dilutions (Log copies) and the y-axis the fluorescence data used for C_T_ determinations in ∆Rn (baseline-corrected normalized fluorescence). Assay was in linear range of Human TTV with R^2^ values (square of the correlation coefficient) of 0.99.

**Figure 2:**
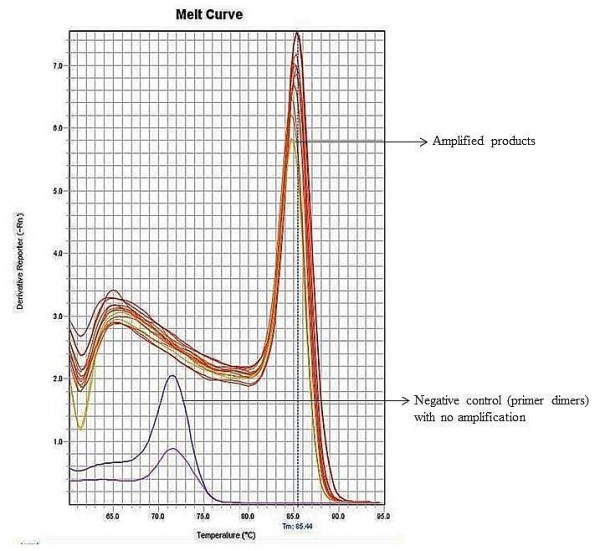
**Melting curve analysis of TTV real-time PCR products.** Y-axis represents the derivative reporter (∆Rn) while x-axis represents the temperature (°C). The figure shows a melting temperature (T_m_) of human TTV PCR products as 85.4°C with no amplifications detected in negative controls except primer-dimers.

**Figure 3:**
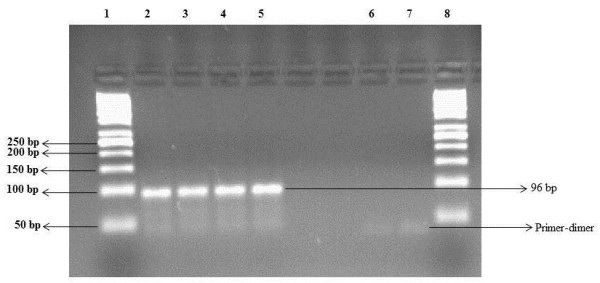
**Agarose gel (3%) electrophoresis of SYBR Green real-time PCR products.** The gel picture shows the presence of primer-dimers in negative control (Lane 6 and Lane 7) and specific TTV amplified products (96 bp) in Lanes 2, 3, 4 and 5. Lane 1 and Lane 8 contained 50 bp DNA ladder.

The assay was validated with serial dilutions of standards ranging from 100 to 10^10^ copies per reaction. The dual sets of primers used showed satisfactory amplification on four different days with assay efficiencies in the range of 81.8% - 82.9% (slopes of the standard curve −3.81 to −3.85). The assay was linear in the range of standards used (co-efficient of regression, R^2^) of 0.99 (Figure 1), which indicates a good correlation between viral copy numbers and cycle threshold (C_T_) values. The lower limit of quantification was determined as 100 copies/reaction (equivalent to 3000 copies/mL of plasma) with an accuracy of 100 ± 5% and intra and inter-day coefficient of variations below 4% (Table 1). The observed melting curve (Melting temperature™: 85.01°C ± 0.27 (mean ± SD; Figure 2) that was clearly different from the melting curve of primer-dimers (T_M_ ranging from 71.1 – 72.9°C) as well as the purity of the TTV-specific 96 bp amplicon in the samples that contained TTV DNA (Figure 3) confirmed the high specificity of the PCR.

**Table 1:** SYBR Green real-time PCR assay for TTV using two independent standards series

**Plasmid – standards**	**Day**	**C**_**T**_Mean ± SD (n=2)*	**Intra-day precision**	**Inter-day precision**	**Intra-day accuracy**	**Inter-day accuracy**
10 × 10^9^	1	4.11 ± 0.09	2.22	0.74	99.9	100.1
	2	4.17 ± 0.08	2.03		99.8	
	3	4.11 ± 0.09	2.36		99.7	
	4	4.09 ± 0.02	0.50		100.2	
10 × 10^8^	1	7.14 ± 0.07	1.10	0.92	102	102.3
	2	7.24 ± 0.22	3.10		101.8	
	3	7.08 ± 0.06	0.88		102.1	
	4	7.11 ± 0.10	1.53		102.3	
10 × 10^7^	1	11.15 ± 0.05	0.45	0.85	101.2	101.4
	2	11.03 ± 0.16	1.49		101.6	
	3	11.26 ± 0.12	1.07		100.8	
	4	11.19 ± 0.06	0.58		101.1	
10 × 10^6^	1	15.21 ± 0.03	0.20	0.31	99.8	100.2
	2	15.10 ± 0.02	0.15		100.1	
	3	15.19 ± 0.01	0.11		100	
	4	15.19 ± 0.003	0.02		99.9	
10 × 10^5^	1	19.15 ± 0.009	0.04	0.28	98.5	98.9
	2	19.03 ± 0.06	0.35		98.8	
	3	19.14 ± 0.02	0.10		98.9	
	4	19.11 ± 0.02	0.12		98.6	
10 × 10^4^	1	22.89 ± 0.009	0.03	0.30	97.9	97.8
	2	22.86 ± 0.09	0.43		97.5	
	3	22.98 ± 0.13	0.57		97.9	
	4	23.00 ± 0.05	0.25		96.8	
10 × 10^3^	1	26.95 ± 0.06	0.24	0.22	94.2	94.3
	2	26.87 ± 0.04	0.17		93.9	
	3	26.95 ± 0.005	0.01		95.3	
	4	27.02 ± 0.13	0.50		92.9	
10 × 10^2^	1	30.83 ± 0.16	0.52	0.35	89.6	88.2
	2	30.89 ± 0.08	0.26		87.2	
	3	31.08 ± 0.03	0.12		88.5	
	4	30.97 ± 0.13	0.42		86.6	
10 × 10^1^	1	34.29 ± 0.30	0.89	0.66	86.3	92.7
	2	33.78 ± 0.57	1.70		93.8	
	3	33.93 ± 0.77	2.29		97.8	
	4	33.87 ± 0.56	1.65		92.3	

*2 independent 10-fold dilution series (n=2) of plasmid standards.

SYBR Green Real-time PCR quantification assay was reproducible with good inter (co-efficient of variation (CV): 0.22 to 1.23%), and intra-day (CV: 0.01 to 3.10%) precision (Table 1). The calculated standard copy numbers were accurate with intra-day and inter-day accuracies in the range of 86.3 to 102.3% and 88.2 to 102.3%, respectively (Table 1). We also observed good reproducibility of the assay when standards were run in triplicates on two different days with intra- and inter-assay precisions of 0.08 to 3.93% and 0.45 to 2.69%, respectively. The intra and inter-day accuracies were 83.8 to 102.1% and 84.3 to 101.3%, respectively (Table 2). Furthermore, our assay yielded similar TTV titers (± 10% variation) in positive controls (4.75 log copies/mL and 2.94 log copies/mL) that were kindly provided by Maggi’s group, Pisa, Italy.

**Table 2:** SYBR Green PCR assay for TTV using single series of plasmid standards

**Plasmid standards**	**Day**	**C**_**T**_Mean ± SD (n=3)*	**Intra-day precision**	**Inter-day precision**	**Intra-day accuracy**	**Inter-day accuracy**
10 × 10^9^	1	4.21 ± 0.02	0.50	0.45	99.6	99.6
	2	4.18 ± 0.01	0.39		99.6	
10 × 10^8^	1	7.59 ± 0.05	0.75	0.65	101.3	101.3
	2	7.52 ± 0.05	0.78		101.2	
10 × 10^7^	1	11.74 ± 0.05	0.45	1.10	100.8	100.8
	2	11.56 ± 0.01	0.14		100.8	
10 × 10^6^	1	15.83 ± 0.04	0.28	1.22	100.4	100.4
	2	15.56 ± 0.01	0.08		100.4	
10 × 10^5^	1	19.75 ± 0.05	0.26	1.39	100.6	100.7
	2	19.37 ± 0.01	0.10		100.8	
10 × 10^4^	1	24.15 ± 0.03	0.12	1.52	98.1	98.4
	2	23.64 ± 0.02	0.10		98.6	
10 × 10^3^	1	28.40 ± 0.03	0.12	1.64	95.3	95.8
	2	27.75 ± 0.05	0.19		96.4	
10 × 10^2^	1	33.21 ± 0.20	0.62	1.51	83.8	84.3
	2	32.51 ± 0.06	0.21		84.8	
10 × 10^1^	1	35.59 ± 0.39	1.11	0.67	102.1	99.0
	2	35.25 ± 0.06	0.17		95.7	

*Tenfold dilutions run in triplicates.

**Figure 4:**
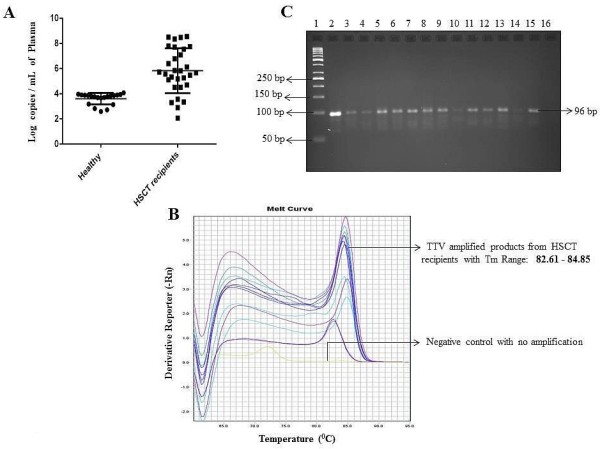
**TTV DNA in healthy individuals (n=20) and in HSCT recipients (n=30) by SYBR Green assay.A** represents the log copies per mL of plasma for each healthy individual and HSCT recipient tested. **B** shows the melting curves for HSCT recipients and **C** shows 3% agarose gel picture with 50 bp DNA ladder in Lane 1, Lane 2 contained positive control (plasmid vector containing TTV insert), Lane 3 - Lane 15 shows the presence of TTV specific PCR products (96 bp) from HSCT recipients while Lane 16 contains the negative control.

In order to check the robustness of the SYBR Green qPCR assay, we measured TTV titers in plasma samples of 20 healthy individuals and 30 HSCT recipients. Variations in TTV loads in terms of log copy numbers of TTV genomes per mL of plasma were found to be in the range of 2.5 – 4.07 log copies/mL (Figure 4A) with a C_T_ (mean ± SD) of 33.8 ± 1.77 for healthy individuals and 2.06 – 8.49 log copies/mL (Figure 4A) with a C_T_ (mean ± SD) of 24.3 ± 1.04 for HSCT recipients. In addition, we observed the differences in melting curves for HSCT recipients (T_M_ ranging from 82.61°C-84.85°C; Figure 4B) which might be due to possible sequence heterogeneity of human TTV strains, with the presence of TTV specific products (96 bp) on 3% agarose gel electrophoresis (Figure 4C).

Several TTV studies using TaqMan chemistry have reported varying levels of sensitivity, ranging from 120 to 1000 copies/mL for different types of clinical specimens [5,14,15,17-22], which may be the result of differences in the primers used. Although sequence heterogeneity in TTV is high with some variants only sharing 50% of nucleotides [23,24], certain conserved regions can be chosen for primer design in order to amplify more than one subtype of TTV [25]. Focosi *et al*. [5] and Maggi *et al*. [19] used probes directed against the conserved portion of untranslated region (UTR), while others used probes specific for highly conserved region of ORF2 and ORF1 of TTV [14,15,17].

**Figure 5:**
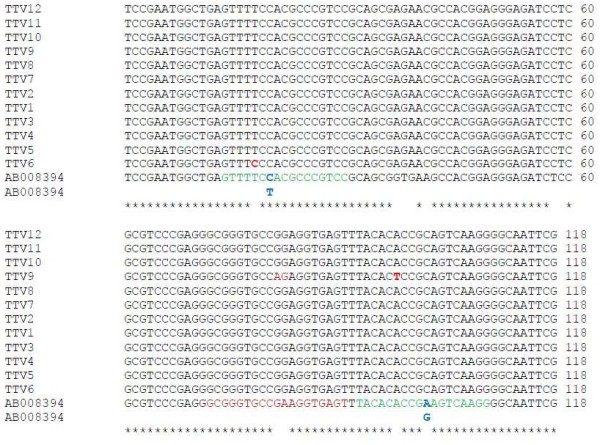
**Sequence alignments of 12 separate TTV clones from a single patient with TTV sequence (AB008394).** The figure shows forward and reverse primers location highlighted in green along with base changes highlighted in blue that were used in dual set of primers for SYBR Green assay and other base changes detected in primer region are highlighted in red. Probe region used previously for TaqMan [14] highlighted in brown.

For our qPCR protocol we used the primers described by Moen *et al.*[14] that differentiate between TTV and TTV-like mini virus (TLMV). Although TaqMan based assays may be somewhat more sensitive, we opted for SYBR Green assay using a primer pair rather than TaqMan technology, which uses two primers and one probe, and is probably more prone to the problem of variable amplification efficacy of strains differing for single nucleotides. Indeed, in our sequencing results for 12 separate clones (Figure 5) from one single patient we observed sequence heterogeneity of TTV in the region that has been used for TaqMan probe [14]. In general, SYBR Green methodology may be more suitable for viral studies where sequence heterogeneity is high in comparison to TaqMan probe-based assays which require high sequence identity for successful probe binding to avoid frequent variable results [14]. In addition, the assay’s threshold of 3000 copies/mL of plasma may be low enough for monitoring TTV in immune deficient patients.

## Conclusion

This report describes the validation of a SYBR Green assay for quantification of TTV viral load in human plasma samples. The developed assay was accurate with satisfactory efficiency, reproducibility in the range of 100-10^10^ copies/ reaction. This simple assay can be used in studies assessing TTV plasma loads as a marker of immune reconstitution. A prospective study is currently ongoing in our institution to validate the correlation of TTV titer and the immune status post HSCT.

## Methods

### Reagents and consumables

QIAamp® MinElute virus spin kit for DNA extraction, QIAprep® Spin Miniprep kit for plasmid extraction and QIAquick® PCR purification kit were obtained from Qiagen, Germany. TA cloning kit dual promoter (pCRII) with One Shot TOP10F`competent cells and ampicillin were obtained from Invitrogen, San Diego, California. DNA Taq polymerase, *BamHI* and *EcoRI* restriction enzymes were obtained from New England BioLabs, USA. SYBR®-Green PCR master mix, 96 well MicroAmp® fast optical reaction plates (0.1 mL capacity) and MicroAmp® optical adhesive films for real-time PCR assay were obtained from Applied Biosystems, Fostercity, CA. All the experiments were performed on StepOnePlus®-Real Time PCR Systems by Applied Biosystems, Fostercity, CA. For amplification of human torque teno virus (TTV), a set of primer pairs described previously were used (Table 3). Primers were made according to the reference strain of TTV genome TA 278 (Gen Bank acc. No. AB008394) and were synthesized by Microsynth (Switzerland) at a scale of 0.2 μmol. DNA ladders, MgCl_2_, dNTP’s and buffers were obtained from Fermentas Life sciences, Germany.

**Table 3:** Oligonucleotide primers used for human torque teno virus (TTV)

**Primers ID**	**Sequence (5` to 3`)**	Nucleotide position*	**Reference**
TTVf	TCCGAATGGCTGAGTTT	102-118	Moen EM *et al.*[19]
TTVr	CGAATTGCCCCTTGACT	219-203	
TTV-F1	GTTTTCTACGCCCGTCC	115-131	
TTV-F2	GTTTTCCACGCCCGTCC	115-131	
TTV-R1	CCTTGACTCCGGTGTGTAA	210-192	
TTV-R2	CCTTGACTTCGGTGTGTAA	210-192	

*According to the reference strain of TTV genome TA 278 (Gen Bank acc. No. AB008394).

### Samples and DNA extraction

Blood samples (5 mL) collected in EDTA tubes from 20 healthy adult volunteers and 30 randomly selected adult HSCT recipients were centrifuged at 900 g for 10 minutes to separate plasma which was immediately frozen at -20°C until used for DNA extraction. Two independent DNA extractions were performed for each of the healthy individuals along with one independent DNA extraction for HSCT recipients, each from 200 μl of plasma using QIAamp MinElute Virus Spin kit according to the manufacturer’s recommendations. DNA was eluted in 30 μL of Milli-Q water. All extracted DNA samples were stored at -20°C until the analysis. The study protocol was approved by the institution’s ethics committee and healthy donors and HSCT recipient’s samples were used after obtaining informed consent.

### Construction of plasmids for standards preparation

A region of 119 bp PCR fragment of TTV genome was amplified using primers TTVf and TTVr (Table 3). Resulting amplicon was purified using QIAquick PCR Purification kit, quantified by spectrophotometer and then cloned into the TA cloning vector. The resulting plasmid was transformed into One Shot TOP10F` competent cells according to instructions provided by the manufacturer. Twelve, isolated colonies of transformed competent cells from solid luria-bertani medium containing ampicillin (100 μg/mL) were subjected for TTV insert confirmation. Each individual colony was suspended separately into 3 mL of liquid luria-bertani medium containing 100 μg/mL of ampicillin for overnight in a shaking incubator at 37°C with a speed of 225 rpm. Following overnight incubation, plasmids purification was done using QIA prep Spin Miniprep kit according to manufacturer’s instructions. Restriction enzyme digestion with *EcoRI* for the purified plasmids was done to confirm the presence of cloned TTV insert (119 bp) on 1.5% agarose gel electrophoresis (data not shown). TTV insert (119 bp) cloned into TA vector were sequenced for all the 12 separate clones using M-13 forward and reverse primers and confirmed by aligning with the TTV sequence (Gen Bank acc. no. AB008394). This plasmid with TTV inserts was linearized with *BamHI* enzyme and then used for preparation of standards in serial 10 fold dilutions from 10×10^9^ copies to 20 copies/μL.

### Absolute quantification of TTV DNA

PCR reaction for absolute quantification of TTV DNA using SYBR Green in a 25 μL reaction is as follows: each reaction contained 12.5 μL SYBR Green PCR master mix, 5 μL of template (serial 10 fold dilutions of the linearized plasmid standards or/ extracted DNA from the plasma samples of healthy blood donors), 1.25 μL (500 nm) of each primer (TTVF-1, TTVF-2, TTVR-1, TTVR-2) and 2.5 μL of Milli-Q water. The cycling conditions included initial activation of AmpliTaq Gold DNA polymerase (present in SYBR Green master mix) for 10 minutes at 95°C. The subsequent PCR conditions consisted of 40 cycles of denaturation at 95°C for 15 seconds and annealing and extension at 60°C for 1 minute per cycle. After real-time data acquisition, the cycle threshold value was calculated by determining the point at which the fluorescence exceeds an arbitrary threshold limit. Standards with known TTV DNA copies were prepared in two independent serial dilutions and were run in the range of 100 copies to 10×10^9^ copies on four non-consecutive days to evaluate biological, inter, intraday variability and accuracy of the assay. In addition, a series of standards from one serial dilution were also run in triplicates on two different days to evaluate the intra-day and inter-day variations. The variability of the assay was evaluated by comparing the C_T_ values run on the same day (intra-day) and on different days (inter-day) and was represented as co-efficient of variations (CV). Accuracy was calculated by taking the ratio of back calculated copies from the standard curve to the theoretical copy number of the reactions. Real-time PCR assay for test samples (HSCT recipients) and for biological replicates of each healthy individual were performed with the inclusion of TTV plasmid standards and negative controls in each run. In addition to this, precision of the assay was also checked by running known TTV positive DNA (positive controls with exact log copies/mL). The viral genomic copies per mL of plasma was calculated as described by Huang *et al*. [26] i.e., by multiplying the copies per reaction by a factor of 30 [30 μL extracted DNA/5 μL of template x (1 mL/200 μL plasma)].

### Melting curve analysis for specificity

Following amplification, melting curve or dissociation curve analysis was performed to measure the specificity of the PCR product. The temperature program used for the melting curve analysis was 95°C for 15 seconds followed by 60°C for 1 minute and then 95°C for 15 seconds with ramp rate of +0.3°C/second.

## Competing interests

The authors declare that they have no competing interests.

## Authors’ contributions

AKT and AP performed the experiments. AKT, AP, PHD, CRSU and MA analyzed and contributed to the interpretation of data. MA and ER designed the research. AKT and MA drafted the article. All the authors revised the manuscript critically. All authors read and approved the final manuscript.

## References

[B1] King AMQ, Adams MJ, Carstens EB, Lefkowitz EJ Virus taxonomy: classification and nomenclature of viruses: Ninth Report of the International Committee on Taxonomy of Viruses 2012 1 San Diego: Elsevier Academic Press 329 426

[B2] Hino S Miyata H Torque teno virus (TTV): current status Rev Med Virol 2007 17 45 57 10.1002/rmv.524 17146841

[B3] Bendinelli M Pistello M Maggi F Fornai C Molecular Properties, Biology and Clinical Implications of TT Virus, a Recently Identified Widespread Infectious Agent of Humans Clin Microbiol Rev 2001 14 98 113 10.1128/CMR.14.1.98-113.2001 11148004 PMC88963

[B4] Touinssi M Gallian P Biagini P Attoui H Vialettes B Berland Y Tamalet C Dhiver C Ravaux I De-Micco P TT virus infection: prevalence of elevated viraemia and arguments for the immune control of viral load J Clin Virol 2001 21 135 141 10.1016/S1386-6532(01)00157-3 11378494

[B5] Focosi D Maggi F Albani M Macera L Ricci V Gragnani S Beo SD Ghimenti M Antonelli G Bendinelli M Pistello M Ceccherini-Nelli L Petrini M Torquetenovirus viremia kinetics after autologous stem cell transplantation are predictable and may serve as a surrogate marker of functional immune reconstitution J Clin Virol 2010 47 189 192 10.1016/j.jcv.2009.11.027 20034850

[B6] Okamoto H Nishizawa T Kato N Ukita M Ikeda H Iizuka H Miyakawa Y Mayumi M Molecular cloning and characterization of a novel DNA virus (TTV) associated with posttransfusion hepatitis of unknown etiology Hepatol Res 1998 10 1 16 10.1016/S1386-6346(97)00123-X

[B7] Mushahwar IK Erker JC Muerhoff AS Leary TP Simons JN Birkenmeyer LG Chalmers ML Pilot-Matias TJ Desai SM Molecular and biophysical characterization of TT virus: Evidence for a new virus family infecting humans Proc Natl Acad Sci USA 1999 96 3177 3182 10.1073/pnas.96.6.3177 10077657 PMC15915

[B8] Miyata H Tsunoda H Kazi A Yamada A Khan MA Murakami J Kamahora T Shiraki K Hino S Identification of a novel GC-rich 113-nucleotide region to complete the circular, single-stranded DNA genome of TT virus, the first human circovirus J Virol 1999 73 3582 3586 10196248 10.1128/jvi.73.5.3582-3586.1999 PMC104131

[B9] Okamoto H Nishizawa T Ukita M Takahashi M Fukuda M Iizuka H Miyakawa Y Mayumi M The entire nucleotide sequence of a TT virus isolate from the United States (TUS01): Comparison with reported isolates and phylogenetic analysis Virology 1999 259 437 448 10.1006/viro.1999.9769 10388667

[B10] Okamoto H Nishizawa T Takahashi M Asabe S Tsuda F Yoshikawa A Heterogeneous distribution of TT virus of distinct genotypes in multiple tissues from infected humans Virology 2001 288 358 368 10.1006/viro.2001.1097 11601907

[B11] Okamoto H Takahashi M Nishizawa T Ukita M Fukuda M Tsuda F Miyakawa Y Mayumi M Marked genomic heterogeneity and frequent mixed infection of TT virus demonstrated by PCR with primers from coding and noncoding regions Virology 1999 259 428 436 10.1006/viro.1999.9770 10388666

[B12] Takahashi K Hijikata M Samokhvalov EI Mishiro S Full or near full length nucleotide sequences of TT virus variants (types SANBAN and YONBAN) and the TT virus-like mini virus Intervirology 2000 43 119 123 10.1159/000025034 10971131

[B13] Muljono DH Nishizawa T Tsuda F Takahashi M Okamoto H Molecular epidemiology of TT virus (TTV) and characterization of two novel TTV genotypes in Indonesia Arch Virol 2001 146 1249 1266 10.1007/s007050170089 11556704

[B14] Moen EM Sleboda J Grinde B Real-time PCR methods for independent quantitation of TTV and TLMV J Virol Methods 2002 104 59 67 10.1016/S0166-0934(02)00039-3 12020793

[B15] Pistello M Morrica A Maggi F Vatteroni ML Freer G Fornai C Casula F Marchi S Ciccorossi P Rovero P Bendinelli M TT virus levels in the plasma of infected individuals with different hepatic and extrahepatic pathology J Med Virol 2001 63 189 195 10.1002/1096-9071(20000201)63:2<189::AID-JMV1014>3.0.CO;2-H 11170056

[B16] Christensen JK Eugen-Olsen J Sorensen M Ullum H Gjedde SB Pedersen BK Nielsen JO Krogsgaard K Prevalence and prognostic significance of infection with TT virus in patients infected with human immunodeficiency virus J Infect Dis 2000 181 1796 1799 10.1086/315440 10823787

[B17] Kato T Mizokami M Mukaide M Orito E Ohno T Nakano T Tanaka Y Kato H Sugauchi F Ueda R Hirashima N Shimamatsu K Kage M Kojiro M Development of a TT virus DNA quantification system using real-time detection PCR J Clin Microbiol 2000 38 94 98 10618070 10.1128/jcm.38.1.94-98.2000 PMC86028

[B18] Takahashi M Asabe S Gotanda Y Kishimoto J Tsuda F Okamoto H TT Virus Is Distributed in Various Leukocyte Subpopulations at Distinct Levels, with the Highest Viral Load in Granulocytes Biochem Biophy Res Commun 2002 290 242 248 10.1006/bbrc.2001.6183 11779160

[B19] Maggi F Fornai C Vatteroni ML Siciliano G Menichetti F Tascini C Specter S Pistello M Bendinelli M Low Prevalence of TT Virus in the Cerebrospinal Fluid of Viremic Patients With Central Nervous System Disorders J Med Virol 2001 65 418 422 10.1002/jmv.2051 11536254

[B20] Rotundo R Maggi F Nieri M Muzzi L Bendinelli M Pini Prato GP TT virus infection of Periodontol Tissues: A controlled Clinical and Laboratory Pilot Study J Periodontol 2004 75 1216 1220 10.1902/jop.2004.75.9.1216 15515336

[B21] Pinho-Nascimento CG Leite JPG Niel C Mendes LD Torque Teno Virus in Fecal Samples of Patients With Gastroenteritis: Prevalence, Genogroups Distribution, and Viral Load J Med Virol 2011 83 1107 1111 10.1002/jmv.22024 21503927

[B22] Vasilyev EV Trofimov DY Tonevitsky AG Ilinsky VV Korostin DO Rebrikov DV Torque Teno Virus (TTV) distribution in healthy Russian population Virol J 2009 6 134 137 10.1186/1743-422X-6-134 19735552 PMC2745379

[B23] Tanaka Y Primi D Wang RY Umemura T Yeo AE Mizokami M Alter HJ Shih JW Genomic and molecular evolutionary analysis of a newly identified infectious agent SEN virus and its relationship to the TT virus family J Infect Dis 2001 183 359 367 10.1086/318091 11133366

[B24] Hallett RL Clewley JP Bobet F McKiernan PJ Teo CG Characterization of a highly divergent TT virus genome J Gen Virol 2000 81 2273 2279 10950985 10.1099/0022-1317-81-9-2273

[B25] Takahashi K Iwasa Y Hijikata M Mishiro S Identification of a new human DNA virus TTV-like mini virus, TLMV intermediately related to TT virus and chicken anemia virus Arch Virol 2000 145 979 993 10.1007/s007050050689 10881684

[B26] Huang YW Dryman BA Harrall KK Vaughn EM Roof MB Meng XJ Development of SYBR green-based real-time PCR and duplex nested PCR assays for quantitation and differential detection of species- or type-specific porcine Torque teno viruses J Virol Methods 2010 170 140 146 10.1016/j.jviromet.2010.09.018 20863859

